# Single-Session Combined Anterior-Posterior Approach for Treatment of Ankylosing Spondylitis with Obvious Displaced Lower Cervical Spine Fractures and Dislocations

**DOI:** 10.1155/2017/9205834

**Published:** 2017-01-04

**Authors:** Baohui Yang, Teng Lu, Haopeng Li

**Affiliations:** Department of Orthopaedics, Second Affiliated Hospital of Xi'an Jiaotong University, Xi'an 710004, China

## Abstract

For patients with AS and lower cervical spine fractures, surgical methods have mainly included the single anterior approach, single posterior approach, and combined anterior-posterior approach. However, various surgical procedures were utilized because the fractures have not been clearly classified according to presence of displacement in these previous studies. Consequently, controversies have been raised regarding the selection of the surgical procedure. This study retrospective analysis was conducted in 12 patients with AS and lower cervical spine fractures and dislocations and explored single-session combined anterior-posterior approach for the treatment of AS with obvious displaced lower cervical spine fractures and dislocations which has demonstrated advantages such as good stabilization, satisfied fracture healing, and easy postoperative cares. However, to some extent, the difficulty and risk of this approach should be considered. Attention should be paid to the prevention of perioperative complications.

## 1. Introduction

Ankylosing spondylitis (AS) is chronic nonspecific inflammatory disease, involving mainly the axial skeleton [1]. Typical AS initially presents with sacroiliitis and gradually develops to affect the intervertebral disc and paravertebral ligament and then causes bamboo spine. Minor trauma may cause spine fracture or dislocation [2] because of decreased elasticity caused by ossification of the intervertebral disc and paravertebral ligament, decreased antishock capability, and osteoporosis [2]. The lower cervical spine is the junction site between the relatively rigid thoracic spine and the flexible cervical spine. Thus, fractures are more common at this site [3]. Fractures often involve 3 columns and cause a very unstable spine. Caron et al. [4] described the classification of this type of fracture. However, their classification was not significant for surgical treatment. Meanwhile, the surgical treatment for AS with lower cervical spine fracture has been reported in several studies [5–7]. However, various surgical procedures were utilized because the fractures have not been clearly classified according to presence of displacement in these previous studies. Consequently, controversies have been raised regarding the selection of the surgical procedure. This study aimed to investigate the efficacy and complications of a single-session combined anterior-posterior approach for treating AS with obvious displaced lower cervical spine fractures and dislocations.

## 2. Materials and Methods

### 2.1. Ethics Statement

This study was approved by the Second Affiliated Hospital of Xi'an Jiaotong University ethics committee.

### 2.2. General Data

A retrospective analysis was conducted in 12 patients with AS and lower cervical spine fractures and dislocations who were admitted and treated in our department between January 2009 and November 2014 (inclusion criteria: patients with obvious displaced fractures across either the intervertebral disc or vertebral body; exclusion criteria: patients without obvious displaced fractures across the intervertebral disc or vertebral body). Of these patients, 6 cases were diagnosed with C7-T1 fractures, 5 cases were diagnosed with C6-C7 fracture, and 1 case was diagnosed with C5-C6 fractures. There were 10 males and 2 females. Their average age was 55.42 ± 6.54 years. All 12 patients had been diagnosed with AS before surgery, of which 5 patients were on medication for AS. The mechanism of injury was traffic accidents in 6 cases, falls in 4 cases, a bumping injury in 1 case, and injury from a falling heavy object in 1 case. Imaging data revealed fractures and dislocations involving the cervical spine in all cases, of which 2 cases were accompanied by thoracolumbar spine fractures. The preoperative ASIA classification was as follows: 2 cases with grade A, 7 cases with grade B, and 3 cases with grade C (Table 1). Preoperative skull traction was performed in 8 cases but could not be performed in the other 4 cases due to the patients' inability to lie on their backs.

### 2.3. Clinical Diagnosis

All patients were admitted to the hospital and received the following routine laboratory tests, including ESR, C-reactive protein (CRP), antistreptolysin O (anti-“o”), and HLA-B27; preoperative studies such as anteroposterior and lateral views of X-rays of the cervical spine, CT scan with 3-dimensional reconstruction, and MRI were completed. Finally, the diagnosis was confirmed according to the patient history, symptoms, physical signs, and the above-mentioned laboratory and imaging data using New York criteria [8]. Patients with AS and nondisplaced fracture were excluded.

### 2.4. Surgical Procedure

Surgery via the anterior approach was performed first; then, the patients were placed in the prone position for surgery via the posterior approach. For the anterior approach, the patients were placed in the supine position with elevation of the shoulder by a surgical pillow; the right-neck oblique approach was used to expose the longitudinal ligament and fracture site of the cervical spine between the visceral and vascular sheath. Based on the defect of the fracture site or decompression condition, reduction, bone grafting or subtotal vertebral body resection, fusion, and internal fixation could be performed. For the posterior approach, the patients were placed in the prone position. The posterior middle approach was used to strip the periosteum paraspinal muscles to expose the side block and perform internal screw fixation. Decompressive laminectomy was performed if necessary.

### 2.5. Management after Surgery

All patients received routine postoperative care such as infection prevention and antiosteoporosis therapy. The drainage tube was removed within 48 hours after surgery if there were no indications showing leakage of cerebrospinal fluid. On postoperative day 2, a cervical collar was used to protect the cervical spine; the patient may have been asked to sit unsupported or supported by the elevated bedhead.

### 2.6. Postoperative Evaluation

The American Spinal Injury Association (ASIA) classification was used to assess the postoperative neurological function. At 2 months after surgery, X-ray anteroposterior and lateral views of the cervical spine and CT scan could be performed to assess the fracture healing and bone graft fusion (fusion criteria: X-rays reveal a continuous trabecular line across the adjacent vertebral bodies at the fracture site [9]); the following signs of complication were observed: infection, cerebrospinal fluid leak, and fixation-related complications.

## 3. Results

The operative time was 3.6 ± 1.1 hours (range: 2.5–5.5 hour). Intraoperative blood loss was 2500 ± 800 mL (range: 1300–3200 mL). Eight patients underwent intervertebral bone grafting, fusion, and internal fixation via the anterior approach. Four patients underwent corpectomy, bone grafting, fusion, and internal fixation. Long segment fixation and fusion, which at least include 2 upper and lower segments from the injured vertebral body, were performed via the posterior approach.

**Figure 1:**
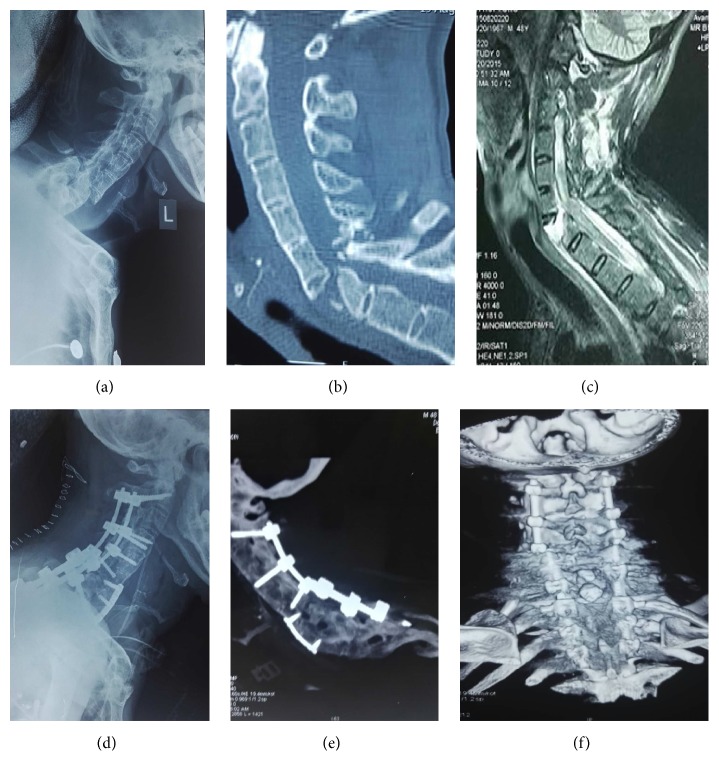
(a) A 48-year-old male was admitted to the hospital because of a car accident. The x-ray shows a typical presentation of AS. (b) Cervical CT scan showing fracture and dislocation of C6-C7, anterior defect of C7, fracture of posterior spinous process, and fracture fragments in the spinal canal. (c) MRI showing a long T2 signal in the intervertebral disc space of C6-C7 and the anterior vertebrae body, a sign of the spinal cord compression. (d, e, f) X-ray and CT scan 3 months after surgery showing good position of the internal fixation and healed fracture.

No injuries of the spinal cord, nerve roots, and vessels caused by screw placement were reported in 12 patients. One patient died, and follow-up was performed in the remaining 11 patients. The follow-up duration was from 10 to 24 months. The average fracture healing time was 3.09 ± 1.14 months (range: 2–5 months). At the end of follow-up, neurological function recovery was assessed according to the spinal cord injury classification. Two patients classified as grade A before surgery were not improved. Of 7 patients classified as grade B before surgery, the function was improved to grade C in 3 patients and to grade D in 2 patients; the function was not improved in 1 patient, and 1 patient died. Of 3 patients classified as grade C, the function was improved to grade D in 2 patients and not improved in 1 patient (Table 1). During follow-up, no complications such as loosening internal fixation or displacement were reported. A typical case is presented in Figure 1.

**Figure 2:**
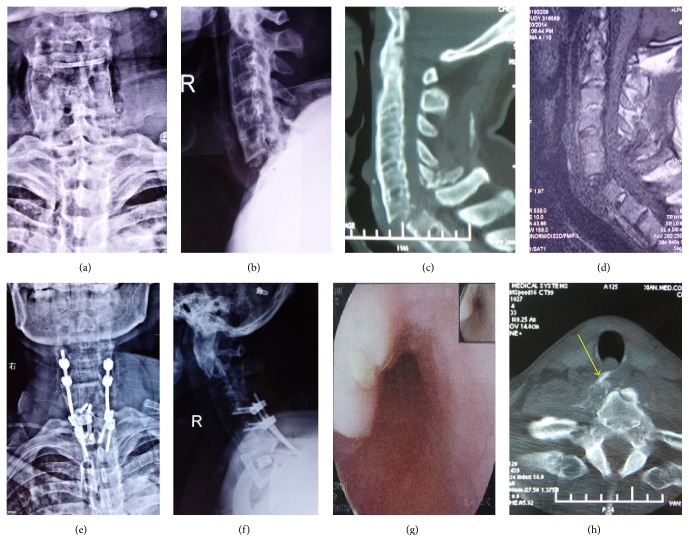
A 53-year-old male was admitted due to sensory-motor disorders of the 4 extremities caused by car accident injuries. (a, b) Anteroposterior and lateral views of an x-ray showing a C6-C7 fracture and dislocation. (c) Sagittal view showing the obviously displaced fracture and dislocation of C6-C7. (d) MRI showing a long T1 signal in the anterior cervical spine. (e, f) Anteroposterior and lateral views showing good position of internal fixation and good reduction. (g) Oesophagoscopy showing a suspicious finding of the tracheoesophageal fistula. (h) Compression of the soft tissue anterior to C6 caused by vertebral fracture fragments (yellow arrow).

In this study, perioperative complications were reported in 3 patients. Among them, isolated postoperative cerebrospinal fluid leakage after posterior surgery was reported in 1 patient who was cured by wound care and symptomatic treatments within 2 weeks. Unilateral recurrent laryngeal nerve injury was reported in 1 patient who presented with hoarseness that subsided at the 1-month follow-up; 1 patient died of tracheoesophageal fistula (Figure 2).

## 4. Discussion

### 4.1. Clinical Characteristics of AS with Lower Cervical Spine Fracture

Long-term AS is usually accompanied by pathological changes such as bony spinal stiffness, vertebral osteoporosis (bamboo spine), and significant ligament calcification of the vertebrae and intervertebral discs [10] and thus causes decreased bone strength in the spine. Therefore, minor trauma can cause fractures of the spine. Furthermore, the lower cervical spine is the junction site between the relatively rigid thoracic spine and the flexible cervical spine. Thus, fractures are more common at this site. Similar to long bone fractures, lower cervical spine fractures often involve the 3 columns and are considered unstable fractures. Due to displacement or dislocation of the upper and lower tips of the fracture, the sharp tips of the fractures can easily damage the spinal cord, the epidural blood vessels, and the oesophagus [11]. Surgical treatment is the first choice for lower cervical spine fracture because patients with AS have pathological features and a biomechanical structure different from that of ordinary patients.

### 4.2. Selection of the Surgical Approach

For patients with AS and lower cervical spine fractures, surgical methods have mainly included the single anterior approach, single posterior approach, and combined anterior-posterior approach.

### 4.3. Single Anterior Approach

The single anterior approach has certain advantages such as less trauma, complete decompression of the anterior column, and a higher fusion rate of the anterior column; many successful cases have been reported [12]. However, in terms of patients with severe fracture displacements, some researchers believe that there may be a risk of plate and screw loosening [13]. The reason may be that the only anterior and middle column of the cervical spine was fixated. The fracture site was the centre of stress; the steel plate cannot withstand the posterior column tension at the position of neck flexion. In addition, the patient's condition is often accompanied by osteoporosis and other pathological changes; therefore, these situations can more easily cause failure of the implanted graft. In recent years, many researchers have suggested using the combined anterior-posterior surgery or posterior approach instead of the single anterior approach [14].

### 4.4. Single Posterior Approach

Biomechanical studies have shown that the stabilization of the cervical spine after posterior fixation and fusion is superior to that of anterior fixation and fusion for treating complete unstable fractures of the cervical spine [15]. However, some researchers have found that the stabilization is insufficient and that the anterior intervertebral injury is difficult to heal. Furthermore, the problem of how to perform anterior decompression cannot be resolved; auxiliary external fixation is usually required after surgery. Moreover, the surgical position is one of the key steps. Patients under anaesthesia are likely to cause secondary spinal cord injury without appropriate protection when changing their position from the supine to the prone position because of poor stabilization of the cervical spine fracture site.

### 4.5. Combined Anterior-Posterior Approach

Given the concurrent decompression of the anterior and posterior column and a 360° full range of spinal fusion, it is doubtless that biomechanical stabilization can be achieved through combined anterior-posterior surgery [16]. More importantly, these patients often have additional, spinal-nerve injuries that may cause a variety of complications; the combined approach can provide exposure to obtain a stronger fixation of fractures, which can benefit patients in early sitting or sitting with support (elevation of the bedhead) and thus reduce pulmonary complications. In terms of treatment for patients with obvious displaced fractures, we suggest utilizing the combined approach to immediately establish spinal stabilization and reduce the likelihood of failed internal fixation, considering they have longer history of the disease, extreme instability of the spine, and osteoporosis. It remains controversial which procedure, anterior approach or posterior approach, should be performed ahead of the other. Some researchers have suggested that preoperative reduction of fractures by skull traction is an indication for initially performing the anterior surgery; and vice versa, posterior surgery should be performed initially if there is the possible presence of articular process interlocking. All 12 patients in this study underwent surgery via the anterior approach followed by surgery via the posterior approach. The fractures were reduced preoperatively in 6 patients and intraoperatively in the other 6 patients. We advocate initially performing anterior surgery because of the following advantages: (1) Initial anterior surgery is convenient for surgical positioning. (2) The reduction via the anterior approach itself is an ideal decompression to avoid the risk of further damage to the spinal cord. Some researchers have argued that it is impossible to perform fracture reduction via the anterior approach due to posterior articular process interlocking. However, we believe that cervical fracture with AS was most likely present as transverse column fracture, which unlikely causes articular process interlocking. Although articular process interlocking was present, a spinal fracture involves a complete break around the spinal cord; thus, its reduction can be performed via either the anterior or posterior approach. The surgical protocol for reduction via the anterior approach is as follows: plate placement on the proximal fracture tip should follow the corpectomy (for decompression purposes), and then reduction should be performed by slightly pulling the distal fracture tip, which has been fixated to the plate by screws. By using this method, we also confirmed that complete reduction can be achieved in patients in whom the fracture reduction cannot be performed before surgery. (3) Temporary fixation via the anterior approach, similar to temporary Kirschner wire fixation during long-bone fracture reduction, was followed by enhanced plate fixation after the posterior surgery, to reduce the secondary risk of fracture displacement caused by the positioning for the posterior surgery. (4) For patients with severe flexion deformity, single anterior plate fixation is impossible due to difficult exposure, especially in the segment below T1. Therefore, short plate fixation via the anterior approach should be performed and followed by posterior surgery, which is considered a good surgical option. However, we must be aware of the drawbacks of the combined anterior and posterior approach, such as massive trauma and increased blood loss and operative time, all of which may increase the risk of perioperative complications.

### 4.6. Prevention of Complications

#### 4.6.1. Before Surgery

Routine preoperative traction was applied. It should be noted that AS fractures are unstable fractures; therefore, preoperative cervical traction differs from the traction for conventional cervical fracture. The direction of the traction for AS fracture should be aligned with the normal cervical curvature before the fracture; the weight for traction should be appropriately reduced. In the patients of our study, the weight for traction was between 2 and 5 Kg. Whether to administer methylprednisolone for early spinal cord injury to reduce the degree of spinal cord injury because of its side effects such as stress ulcers and the aggravation of previously existing diseases is controversial. None of the patients in this study received methylprednisolone treatment. In addition, Lee et al.' study [17] has shown that patients with spinal cord injury should maintain hemodynamic stability; the mean arterial blood pressure should be maintained at 90 mmHg within 48 hours after injury and above 85 mmHg within 1 week after injury.

#### 4.6.2. During Surgery

Surgical positioning is also one of the key steps for the surgery. Basically, special care should be taken regarding any movement of the patients. Neural evoked potentials may be used for monitoring during positioning. The cervical curvature should be consistent with the curvature before injury when placing a head frame or adjusting the operating table. In the prone position (chest pad required), the cephalad side should be higher than the caudal side to reduce spinal pressure and blood loss. In 1 of patients in this study, a “milky substance” was observed between the sheath of the trachea, the oesophagus, and the carotid sheath after opening the cervical platysma via the anterior approach. The milky substance was removed, and none was further observed. Intraoperative exploration did not reveal obvious signs of tracheoesophageal fistula. On postoperative day 3, 400 mL of a malodourous and unclear exudate was discharged from the posterior wound. A gastric endoscopic examination confirmed the diagnosis of tracheoesophageal fistula; subsequently, conservative treatments such as fasting, gastrointestinal decompression, and anti-inflammatory, anti-acid, and intravenous nutrition therapy were initiated; the cervical surgical wound was opened and drained using a gauze strip soaked in hypertonic saline. The patient died of multiple organ failure caused by septic shock on postoperative day 7. The preoperative CT images and 3D reconstruction were reviewed and showed that the soft tissues anterior to the spine were compressed by fracture fragments of C6 and C7 (Figure 2(h)). The postoperative X-ray examination confirmed that the mounting screws were in the appropriate position and did not exceed the anterior edge of the vertebral body. The interpretation for this case is that the patient had a 20-year history of AS and had not received regular treatment. These factors resulted in brittle and rigid fracture fragments, which may easily puncture the oesophagus. In this case, the serious consequences might have been due to the failure to make a proper preoperative diagnosis of oesophageal fistula. Therefore, patients with cervical spine fractures, especially patients with accompanying AS, should complete the preoperative examination to determine whether a preoperative oesophageal fistula is present. For patients who have experienced violent trauma and have obvious fracture displacement, fever and anterior swelling of the neck or for patients who are highly suspected to have oesophageal fistulas, the cervical CTH or MRI images should be carefully reviewed to determine whether there is an abnormal long T1 or T2 signal at the oesophageal segment near to the fractured vertebral spine. Upper gastrointestinal endoscopy and radiography should be used to confirm the diagnosis, if necessary.

The operative approach is consistent with the general approach to the fracture and dislocation of the cervical spine. For those patients with preoperative skull traction and reduction, after the fracture site exposure, they were treated with discectomy or autogenous iliac bone graft in mandibular reconstruction and fixed using steel plates. For those patients with preoperative skull traction and no reduction, they were treated with corpectomy. Firstly, the proximal fracture was fixed using steel plates. After reduction by mild pulling effect, they were treated with autologous bone grafts and distal fracture fixation using screw. In the posterior approach: expose the lateral block and clean up the broken bone tissue and decompress by biting away the yellow ligament by rongeur on the fracture site. The screw fixation of lateral mass and pedicle was involved in two vertebral bodies in the upper and lower part of the fracture site. After removing the laminar outer cortex by rongeur or exposure of the bone graft bed by grinding drill, the broken bone mass and fracture of spinous were processed into granular for bone graft between lamina.

In this study, 1 patient presented with hoarseness, which may be caused by recurrent laryngeal nerve injury. Lower cervical spine fracture is in the path of the recurrent laryngeal nerve. Patients with AS often were presented with neck flexion deformities, which cause difficulty in exposing the surgical field. Consequently, there is a higher risk of recurrent laryngeal nerve injury during surgery. This complication has been reported previously [18]. To avoid possible injury to the recurrent laryngeal nerve, it is recommended to reduce unnecessary traction to the oesophagus and the trachea, to operate beneath the supraomohyoid muscle, and to protect the recurrent laryngeal nerve.

In patients with AS, the dura mater may become thin and adhere to the spinal canal because of loss of the spinal canal adipose tissue. Spinal decompression may result in a dural tear and cerebrospinal fluid leakage. In this study, 1 patient experienced a cerebrospinal fluid leak. If an intraoperative dural tear is identified, a continuous lock suture may be used to close the torn site. In terms of managing a cerebrospinal fluid leak, we performed conventional drainage for 2 weeks and administered enhanced wound dressings. The wound healed smoothly in this patient.

#### 4.6.3. After Surgery

Patients with AS are generally aged and often have accompanying basal lesions. Postoperative complications such as pressure sores, deep vein thrombosis, and pulmonary infection may be present and are considered major causes of patient deaths, for which lung infection is the main cause of mortality [19]. Combined anterior and posterior surgery can increase the stabilization of the cervical spine, facilitate the postoperative care and early functional exercise, and reduce the complications caused by prolonged bed stay. Therefore, for the patients of this study, we recommended early sitting, ambulation, and the application of a venous pump to the lower extremities, to reduce the possibility of postoperative complications.

In summary, a single-session combined anterior-posterior approach for treating AS with obvious displaced lower cervical spine fractures and dislocations has demonstrated advantages such as good stabilization, satisfactory fracture healing, and easy postoperative care. However, to some extent, the difficulty and risk of this approach should be considered. Attention should be paid to prevent perioperative complications.

## Figures and Tables

**Table 1:** General data of the patients.

Number of subjects	Age/years	Sex	Causes of injury	Fracture site	Preoperative ASIA grade	ASIA grade at the end of follow-up	Fracture healing time	Follow-up duration
(1)	61	M	Car accident	C7-T1	B	C	2	12
(2)	57	M	Fall from an electric bicycle	C6-C7	C	D	2	14
(3)	52	M	Car accident	C7-T1	A	A	3	20
(4)	55	F	Fall	C7-T1	C	D	2	10
(5)	44	M	Fall from a bicycle	C6-C7	B	D	5	22
(6)	67	M	Fall on stairs	C5-C6	A	A	3	12
(7)	48	M	Car accident	C7-T1	B	C	3	12
(8)	53	M	Car accident	C6-C7	B	Deceased	—	—
(9)	60	F	Hitting the head on a desk edge	C7-T1	C	C	2	16
(10)	48	M	Car accident	C6-C7	B	B	3	10
(11)	49	M	Car accident	C6-C7	B	C	4	24
(12)	61	M	Being hit by a falling heavy object	C7-T1	B	D	5	20

M: male; F: female.
